# Correction: Park et al. Protective Effects of Nargenicin A1 against Tacrolimus-Induced Oxidative Stress in Hirame Natural Embryo Cells. *Int. J. Environ. Res. Public Health* 2019, *16*, 1044

**DOI:** 10.3390/ijerph22050773

**Published:** 2025-05-14

**Authors:** Cheol Park, Da Hye Kwon, Su Jung Hwang, Min Ho Han, Jin-Woo Jeong, Sang Hoon Hong, Hee-Jae Cha, Su-Hyun Hong, Gi-Young Kim, Hyo-Jong Lee, Suhkmann Kim, Heui-Soo Kim, Yung Hyun Choi

**Affiliations:** 1 Department of Molecular Biology, College of Natural Sciences, Dong-eui University, Busan 47340, Korea; parkch@deu.ac.kr; 2 Department of Biochemistry, Dong-eui University College of Korean Medicine, Busan 47227, Korea; chghl1013@hanmail.net (D.H.K.); hongsh@deu.ac.kr (S.-H.H.); 3 Department of Pharmacy, College of Pharmacy, Inje University, Gimhae 50834, Korea; sama3575@naver.com (S.J.H.); hjlee@inje.ac.kr (H.-J.L.); 4 National Marine Biodiversity Institute of Korea, Seocheon 33662, Korea; mhhan@mabik.re.kr; 5 Nakdonggang National Institute of Biological Resources, Sangju 17104, Korea; jinwooyo@nate.com; 6 Department of Internal Medicine, Dong-eui University College of Korean Medicine, Busan 47227, Korea; shhong@deu.ac.kr; 7 Department of Parasitology and Genetics, Kosin University College of Medicine, Busan 49267, Korea; hcha@kosin.ac.kr; 8 Department of Marine Life Sciences, Jeju National University, Jeju 63243, Korea; immunkim@jejunu.ac.kr; 9 Department of Chemistry, College of Natural Sciences, Center for Proteome Biophysics and Chemistry Institute for Functional Materials, Pusan National University, Busan 46241, Korea; suhkmann@gmail.com; 10 Department of Biological Sciences, College of Natural Sciences, Pusan National University, Busan 46241, Korea; khs307@pusan.ac.kr

In the original publication [1], there was a mistake in Figure 2B,C. This data error occurred due to an unforeseen mistake during the data organization process. The corrected Figure 2 appears below. The authors state that the scientific conclusions are unaffected. This correction was approved by the Academic Editor. The original publication has also been updated.

## Figures and Tables

**Figure 2:**
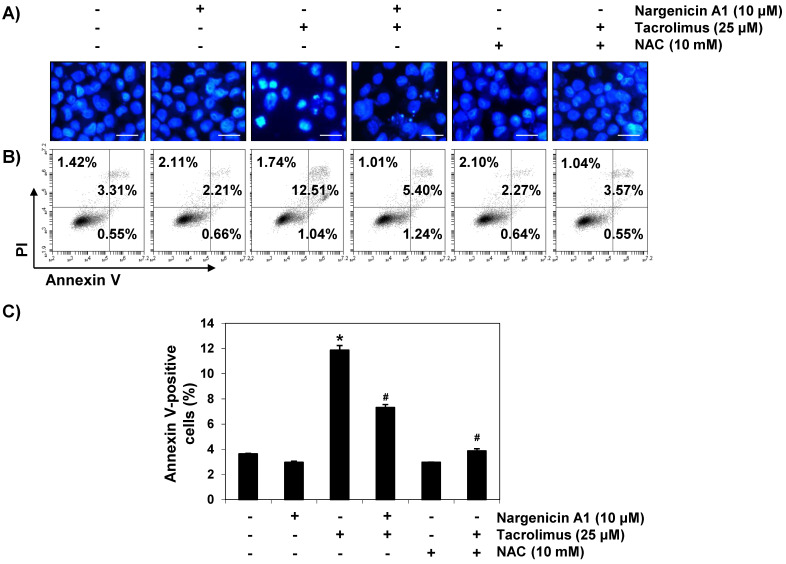
Suppression of tacrolimus-induced apoptosis by nargenicin A1 in HINAE cells. HINAE cells were treated with 10 μM nargenicin A1 or 10 mM NAC for 1 h, and then stimulated with or without 25 μM tacrolimus for 24 h. (**A**) Cells were collected, fixed, stained with 4,6-diamidino-2-phenylindole (DAPI), and photographed under a fluorescence microscope (original magnification, ×400). Scale bar, 50 µm. (**B**,**C**) Cells cultured under the same conditions were collected and stained with fluorescein isothiocyanate (FITC)-conjugated annexin V and propidium iodide (PI) for flow cytometry. (**B**) Results showed necrosis, defined as annexin V-negative and PI-positive cells (lower upper quadrant); early apoptosis, defined as annexin V-positive and PI-negative cells (lower right quadrant); and late apoptosis, defined as annexin V-positive and PI-positive (upper right quadrant) cells. (**C**) Percentages of apoptotic cells were determined by expressing the number of annexin V-positive cells as a percentage of all cells present. Results are presented as the means ± SD of three independent experiments (* *p* < 0.05 versus non-treated controls, # *p* < 0.05 versus tacrolimus-treated cells).
